# Characterization of the gut microbiota in Chinese children with overweight and obesity using 16S rRNA gene sequencing

**DOI:** 10.7717/peerj.11439

**Published:** 2021-06-08

**Authors:** Xiaowei Chen, Dawei Zhang, Haixiang Sun, Fei Jiang, Yan Shen, Pingmin Wei, Xiaobing Shen

**Affiliations:** 1 Key Laboratory of Environmental Medicine Engineering, Ministry of Education, School of Public Health, Southeast University, Nanjing, China; 2 Department of Epidemiology and Health Statistics, School of Public Health, Southeast University, Nanjing, China; 3 Nanjing Municipal Center for Disease Control and Prevention, Nanjing, China

**Keywords:** 16S rRNA gene sequencing, Gut microbiota, Children, Overweight, Obesity, Alpha diversity, Beta diversity

## Abstract

**Background:**

Childhood obesity constitutes a worldwide health problem, and the gut microbiota play extremely important roles in obesity. Herein, we aimed to characterize the gut microbiota in children of normal weight, overweight, and obesity.

**Methods:**

Thirty children of normal weight, 35 who were overweight, and 35 with obesity were enrolled from Nanjing, China. We isolated DNA from fecal samples, and employed 16S rRNA gene sequencing to explore the diversity and composition of gut microbiota.

**Results:**

The operational taxonomic unit (OTU) numbers exhibited a reduction in the gut microbiota abundance with the increase in the body weight. Alpha diversity analysis revealed a sharp decrease in the mean microbial abundance among the three groups (Chao1: *F* = 5.478, *P* = 0.006; observed species: *F* = 7.271, *P* = 0.001; PD whole tree: *F* = 8.735, *P* < 0.001). Beta diversity analysis indicated notable differences in the gut microbial composition between children of normal weight and obesity. However, overweight children had little difference in gut microbiota compared to either children of normal weight or obesity. At the genus level, *Oscillospira* decreased among the three groups (*χ^2^* = 10.062, *P* = 0.001), and *Sutterella* increased (*F* = 4.052, *P* = 0.020). There were many remarkably increased species of gut bacteria in the comparison among three groups, 31 in the normal weight group, 32 in the obese group, and only three species of bacteria were identified in the overweight group. These significantly increased species of gut bacteria may have a close relationship with the progression of obesity.

**Conclusions:**

The abundance of species decreased significantly as the BMI increased. Although the gut microbial composition between children of normal weight and obesity was notably different, due to the changing ratio of some microbial communities, gut microbiota in overweight children showed similarities to that of children with normal weight and obesity.

## Introduction

Globally, the number of people who are overweight and obese is increasing, which poses a great challenge to human health. Data revealed that the prevalence of overweight children, as well as obesity rose in the non-industrialized nations in 2013 from 8.1% to 12.9% and from 8.4% to 13.4% for boys and girls, respectively (Ng et al., 2014). In China, recent years have witnessed a steep rise in obesity to nearly 90 million, ranking China first in the global number of obesity (Di Cesare et al., 2016). This increase was mainly observed among children, and was associated with a sharp escalation in the prevalence of obesity-linked chronic disease and therefore posed a growing burden to society. It is clear that effective measures need to be taken to combat the worldwide obesity epidemic.

Excessive food intake, inadequate physical activity, as well as genetic predisposition constitute the most common causes of obesity. However, studies in recent years suggest that gut microbiota might be closely linked to the occurrence and the progression of obesity. Attention has been newly focused on the gut microbiome’s contribution to obesity since 2005 with a series of studies from Ley et al. (2005, 2006) and Turnbaugh et al. (2006). These studies provided some of the first evidence of a connection between gut microbial composition and obesity. Since then, numerous studies have revealed key insights into the structural, as well as functional composition of the obesity-related gut microbiome (Murugesan et al., 2015; Al-Ghalith, Vangay & Knights, 2015; De Filippo et al., 2010; Dugas et al., 2016; Graham, Mullen & Whelan, 2015; Parekh, Balart & Johnson, 2015).

Gut microbiota is considered the “forgotten organ” or the “second genome” in the human body (Koboziev et al., 2014). There are more than 100 trillion microbes in the gut of a healthy human, and the total number of genes from this microbiota is more than 100 times larger relative to that of the entire human genome. The microbial cell counts in the human gut was approximately 10 times that of human cells (Koboziev et al., 2014; Qin et al., 2010). Normally, there is a dynamic balance between gut microbiota and human body. Once this balance breaks, it results in metabolic disorders and even obesity. Evidence shows that the number of Bacteroidetes decreased while the number of Firmicutes increased in obese rats compared to lean rats under the same diet (Ley et al., 2005). Patrone et al. (2016) indicated that the ratio of Bacteroidetes to Firmicutes in people with obesity was significantly smaller than that in people of normal weight. Turnbaugh et al. (2008) discovered that in the guts of people with obesity, the numbers of Firmicutes increased while that of Bacteroidetes decreased substantially. Moreover, it has been documented that gut microbiota between obese and non-obese persons has different numbers of genomes and abundances (Le Chatelier et al., 2013). In summary, all of these studies revealed a close association in the imbalance of gut microbiota and obesity.

In recent years, the relationship between gut microbiota and obesity has attracted increasing attention. It has been opined that the microbiota continues to develop in children, which may provide unique opportunities for the intervention of the microbiota to promote health or prevent obesity. To establish a baseline understanding of gut microbiota in children, we conducted related studies focusing on the structure, as well as the function of gut microbiota in children. As a result, we applied 16S rRNA gene sequencing to analyze the gut microbial diversity along with the composition in children of normal weight, overweight, and obesity.

## Methods

### Subjects and sample collection

We recruited 100 children of both sexes as study participants, 55 males and 45 females, in Nanjing, China. Strict categorization was conducted to divide subjects into three groups, namely the normal weight group (*n* = 30), the overweight group (*n* = 35), or the obese group (*n* = 35) as per the inclusion criteria, as well as exclusion criteria. Inclusion criteria: (a) The range of age for inclusion was 6 years to 11 years; (b) Normal weight, overweight and obesity were defined as per the “*Body mass index cut-offs for overweight and obesity in Chinese children and adolescents aged 2–18 years*” formulated by the Department of Growth and Development, Capital Institution of Pediatrics (Li et al., 2010), which was displayed in our previous study (Xiaowei et al., 2020) (Table S1); (c) All participants voluntarily joined the study with informed consent from legal guardians. Exclusion criteria: (a) Candidates took antibiotics 4 weeks preceding the study; (b) Candidates had canal disorders or experienced cramps, bloating, diarrhea or constipation in the 4 weeks preceding the study; (c) Candidates suffered from trauma, serious infection or infectious diseases; (d) Candidates had genetic obesity or drug-induced obesity; (e) Candidates had endocrine dyscrasia or metabolic diseases. The study was approved by the ethics committee of Zhongda Hospital, Southeast University, and conducted as per the *Declaration of Helsinki*. Moreover, participation was voluntary, and all legal guardians gave written informed consent. We collected the fecal samples as described previously (Xiaowei et al., 2020).

### DNA extraction and PCR amplification

DNA was extracted from the fecal samples by the TIANamp Stool DNA kit (TIANGEN, China, Cat. DP328) as per instructions given in the manufacturer protocol. The NanoDrop One was employed to determine the DNA concentration, as well as purity. The primers 515F (5′-GTGCCAGCMGCCGCGGTAA-3′) along with 806R (5′-GGACTACHVGGGTWTCTAAT-3′) targeting the bacterial 16S rDNA V4 region were used in the PCR (Shaoming et al., 2017). The PCR amplification conditions constituted: 5 min at 94 °C for initial denaturation, followed by 30 cycles of 30 s denaturation at 94 °C, 30 s annealing at 52 °C, and 30 s extension at 72 °C, and a final 10 min elongation at 72 °C. More operational details of follow-up steps were as previously described (Xiaowei et al., 2020).

### Library preparation and sequencing

Processing of sequencing libraries were performed with the NEBNext^®^ Ultra^™^ DNA Library Prep kit from Illumina^®^. Afterwards, sequencing was done on the Illumina Hiseq2500 platform (Caporaso et al., 2011) for the generation of 250 bp paired-end reads. Assigning of the generated sequences to each sample was done as per their unique barcode and primer, whose maximum allowable errors were set as 2 and 3, respectively. After that, we removed the barcodes, as well as the primers and remained with the clean paired-end reads. Subsequently, the FLASH software (Tanja & Salzberg, 2011) was employed to merge these reads based on the association of the overlap of the paired-end reads. The minimum length of the read that overlapped the read generated from the opposite end of the same DNA fragment was 10 bp, and the maximum admissible error ratio of the overlap region was 0.2. Moreover, the Trimmomatic software (Bolger, Marc & Bjoern, 2014) was employed to perform quality filtering on the spliced sequences to obtain the clean tags.

### OTU cluster and species annotation

Clustering of the dataset into operational taxonomic units (OTUs) was on the basis of a 97% sequence similarity via average neighbor clustering. In each of the representative sequence in OTU, the Greengenes database was utilized based on the Ribosomal Database Project (RDP) classifier algorithm (Cole et al., 2007), and the assign_taxonomy.py script in Quantitative Insights Into Microbial Ecology (QIIME) (Caporaso et al., 2010) was used to annotate taxonomic information. KRONA software (Ondov, Bergman & Phillippy, 2011) was used to compare the phylogenetic relationships of different OTUs by visualizing the data of each sample annotations, and GraPhlAn software (Lesueur et al., 2015) was employed to investigate the species composition, as well as the richness information of the sample with the help of a single sample OTU annotation circle graph. Besides, the OTU representative sequences with the relative richness in the first 50, as well as annotated to the level of the genus were selected for multiple sequence alignment using Mafft software (Kazutaka et al., 2002). With the tree structure established using FastTree software (Price, Dehal & Arkin, 2009), the relative abundance of each OTU, as well as the species annotation data of the representative sequence were integrated by the ggtree software package (Yu et al., 2017) for visual display.

### Diversity and LEfSe analysis

Alpha diversity was applied according to the OTU abundance table, which was comprehensively calculated with QIIME software through 5 indices consisting of the Chao1, observed species, PD whole tree, Shannon, and Simpson indices. Specifically, QIIME software was adopted to calculate a diverse index for the visualization of rarefaction curves, as well as rank-abundance curves using R software (Core et al., 2014). Beta diversity on Unifrac were computed with the QIIME software. Additionally, principal coordinate analysis (PCoA) was conducted to obtain principal coordinates, as well as to visualize complex, multidimensional data, and PCoA analysis was exhibited using QIIME2 and the ggplot2 package (Ginestet, 2011). The UPGMA (Unweighted Pair-group Method with Arithmetic Means) approach based on the upgma_cluster.py script in QIIME was employed to perform sample cluster analysis. Nonmetric multidimensional scaling (NMDS) analysis was done using the vegan package (Dixon, 2003) based on the normalized OTU abundance table. Besides, the linear discriminant analysis (LDA) effect size (LEfSe) assessment was as described previously (Xiaowei et al., 2020).

### Ethics statement

The study was approved by the ethics committee of Zhongda Hospital, Southeast University (approval number: 2017ZDSYLL109-P01), and conducted as per the Declaration of Helsinki. Participation was voluntary and all legal guardians gave written informed consent.

### Statistical analysis

The IBM SPSS 23.0 software was employed to perform all statistical analyses. Shapiro-Wilk test was performed to test the normality of Data distribution. Where distribution was normal, differences were assessed using ANOVA. Where there was non-normal distribution, a Kruskal-Wallis test was used. Test results were considered significant at an alpha of *P* < 0.05.

## Results

### Study population features

Overall, 100 samples were collected for this study from 30 children of normal weight, 35 overweight children and 35 children with obesity. There were no remarkable sex and age differences between the three groups (*P* > 0.05), but differences in BMI were statistically significant (*P* < 0.001) (Table 1).

**Table 1:** Characteristics of the study population.

Variables	Normal weight (*n* = 30) Mean (SD)	Overweight (*n* = 35) Mean (SD)	Obesity (*n* = 35) Mean (SD)
Gender (boys/girls)	14/16	19/16	20/15
Age (years)	9.07 (1.64)	8.69 (1.60)	8.43 (1.46)
BMI (kg/m^2^)	15.23 (0.46)	19.91 (1.76)	24.32 (3.01)*

**Note:**

* *P* < 0.05 asterisks indicate statistical significance.

### Sequencing coverage

**Figure 1:**
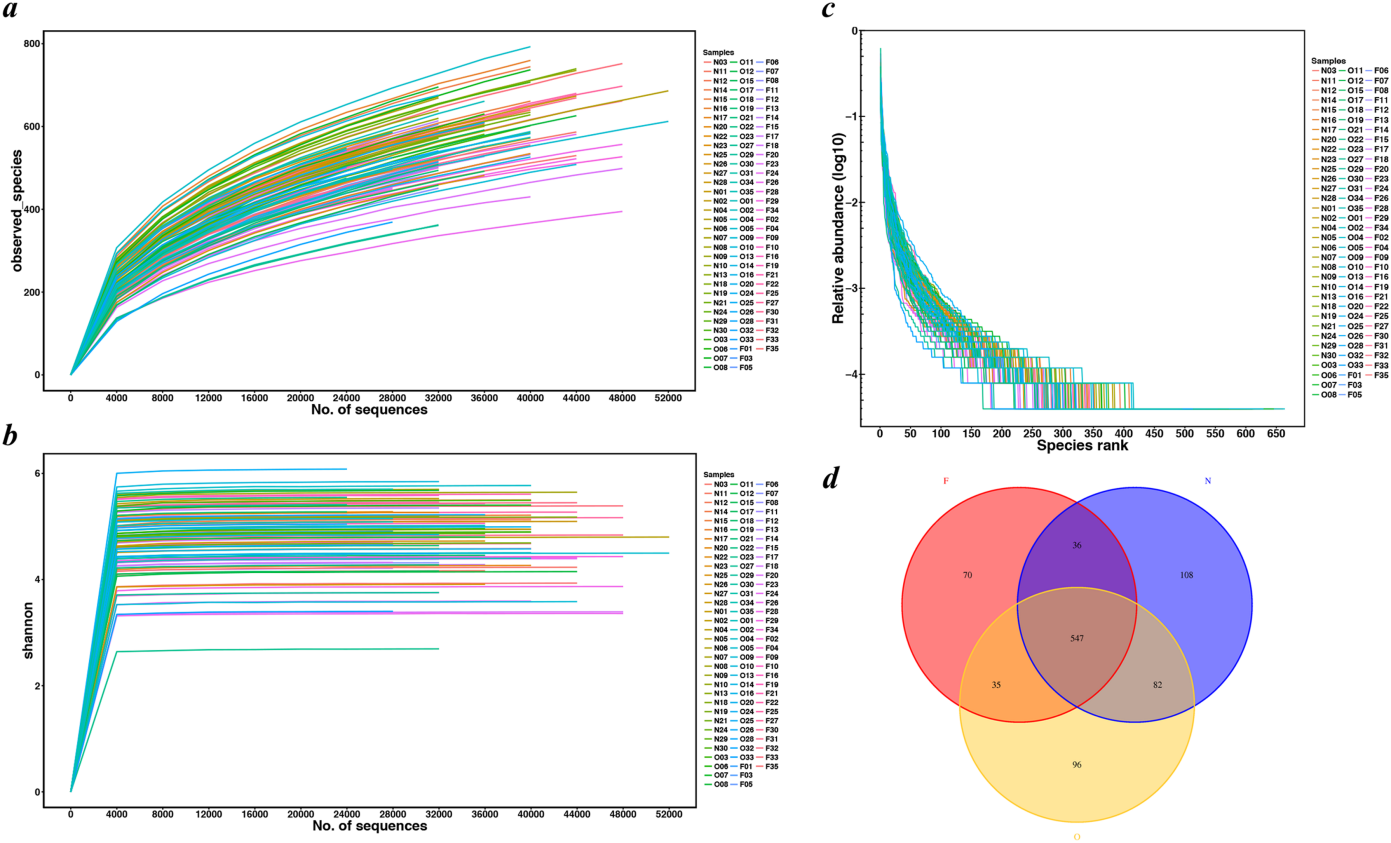
Rarefaction curves and Venn diagram for OTUs calculated. (A) Observed species curves and (B) Shannon curves represent the observed number of species in three study groups. The y-axis indicates the average number of OTUs per sample in each group. Rarefaction curves show the observed species at various sequencing depths. (C) The rank-abundance curve had a steep slope. (D) Red, Yellow and blue represent three study groups. The intersections represent the OTUs shared with the other one or two groups while the single-layer zone represents the number of OTUs specific to each group. (N, normal weight group; O, overweight group; F, obese group).

We extracted a total of 4,482,565 raw sequence reads from the 100 samples, and obtained 3,839,582 high-quality, as well as classifiable 16S rRNA gene sequences following a series of quality filtering. The average sequences number for each sample was 38,396 (ranging from 25,301 to 55,389). Rarefaction curves (Figs. 1A and 1B) did not reach a plateau, which demonstrated that even though deeper sequencing may identify the rare OTUs, the bulky of microbial diversity had been captured. The rank-abundance curve had a steep slope (Fig. 1C). All shared and unique communities among three groups were revealed in the Venn diagram (Fig. 1D). A total of 2,221 OTUs were obtained, of which 547 OTUs were shared by the three groups while 108 genera were specific to the normal weight group, 96 genera to the overweight group and 70 genera to the obese group. These observations demonstrated that the gut microbiota abundance decreased with the increase in body weight.

### Fecal bacterial diversity estimation

**Figure 2:**
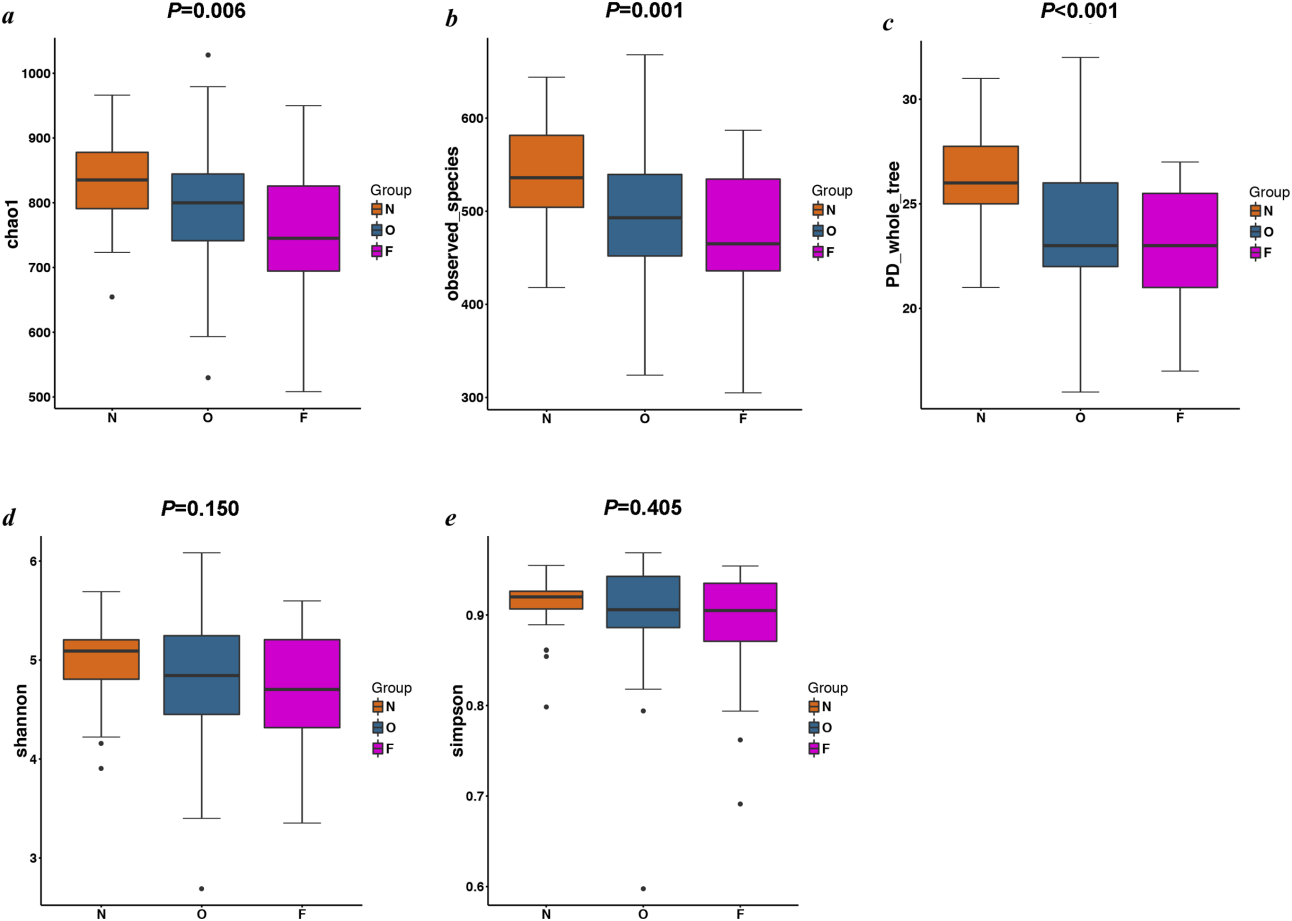
Alpha diversity metrics of OTU-level fecal bacterial communities. (A & B) Boxplots for comparison of species richness (Chao1 index and observed species); (C) Boxplots for comparison of phylogenetic diversity (PD whole tree); (D & E) Boxplots for comparison of species diversity (Shannon index and Simpson index). (N, normal weight group; O, overweight group; F, obese group).

**Figure 3:**
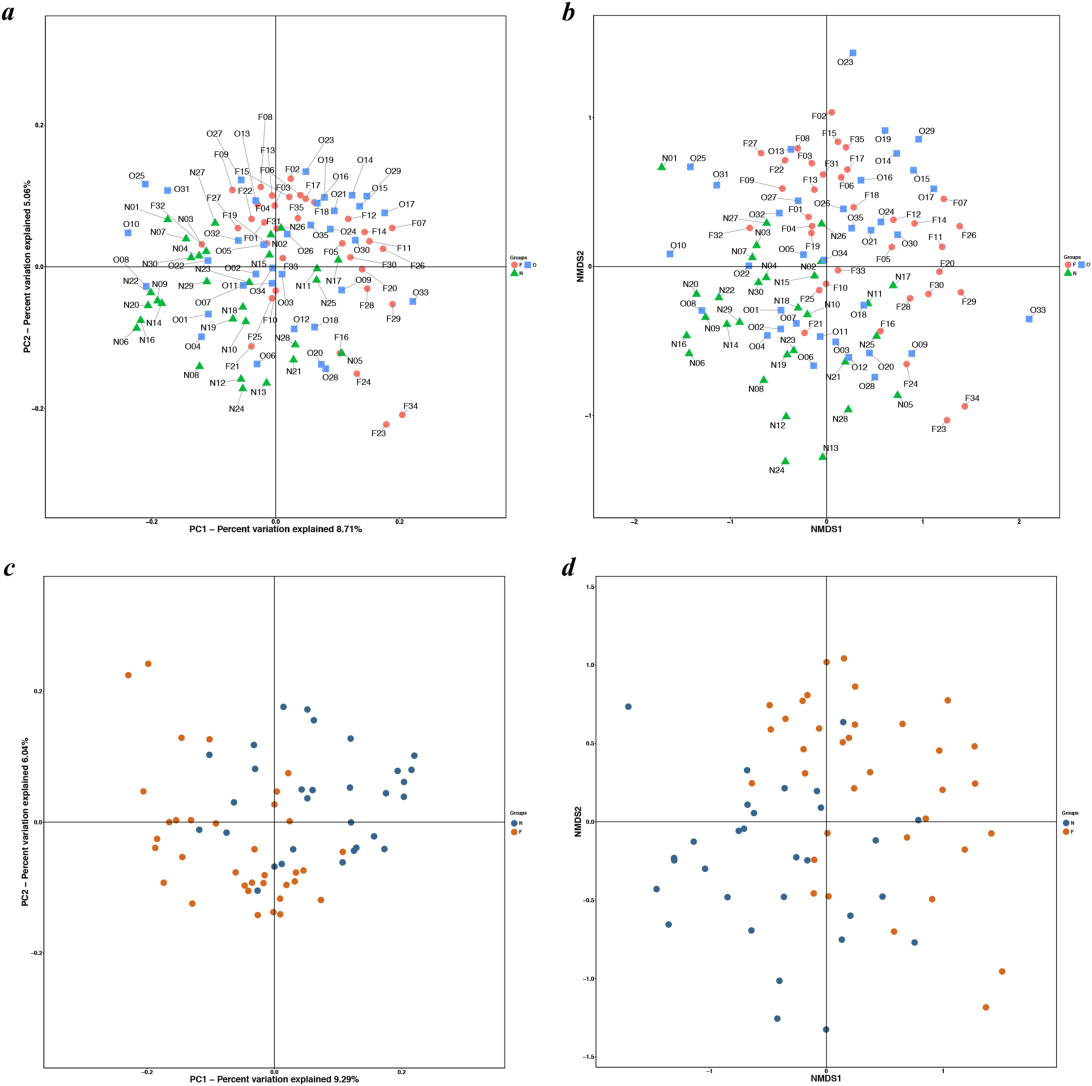
PCoA and NMDS based on the abundance of OTUs. PCoA and NMDS plots showed sample distribution among the three groups (A, B) and between the two groups (C, D) (N, normal weight group; O, overweight group; F, obese group).

The results of the alpha diversity indices showed a remarkable reduction in the mean microbial abundance among the three groups (Chao1: *F* = 5.478, *P* = 0.006; observed species: *F* = 7.271, *P* = 0.001; PD whole tree: *F* = 8.735, *P* < 0.001), but the Shannon index and the Simpson index were not statistically significant (Shannon: *χ*^*2*^ = 3.775, *P* = 0.150; Simpson: *F* = 1.177, *P* = 0.405) (Fig. 2). The similarities among gut microbial communities (beta diversity) were compared via UniFrac analysis, while differences were revealed by NMDS, as well as PCoA according to the abundance of OTUs (Fig. 3). NMDS and PCoA plots did not completely discriminate (no obvious separation) between the overweight group and the other two groups, with partial coincidence points of the normal weight or the obese group (Figs. 3A and 3B). Separation between normal weight group and obesity group was particularly evident when overweight group were excluded, points clustered at the bottom left and the top right denote the gut microbial composition of the normal weight group, as well as the obese group, respectively (Figs. 3C and 3D). Furthermore, we analyzed the significance in the differences in community structures by a non-parametric approach to multivariate analysis of variance (Adonis) and analysis of molecular variance (AMOVA). The results showed that only community structures in the normal weight group and obese group revealed marked differences (Adonis: *SS* = 0.406, *MS* = 0.406, *Fs* = 1.715, *R*^*2*^ = 0.026, *P* = 0.024; AMOVA: *SS* = 0.406, *MS* = 0.406, *Fs* = 1.715, *P* = 0.018; Tables 2 and 3).

**Table 2:** Differences in bacterial community structures among three groups by Adonis.

GroupVS	*Df*	*SS*	*MS*	*Fs*	*R*^*2*^	*P*
N-O-F	2	0.605	0.303	1.289	0.026	0.109
N-O	1	0.222	0.222	0.963	0.015	0.488
N-F	1	0.406	0.406	1.715	0.026	0.024*
O-F	1	0.282	0.282	1.189	0.017	0.25

**Notes:**

* *P* < 0.05 asterisks indicate statistical significance.

Df represents degree of freedom. SS is the total variance, also known as Sums of squared deviations. MS stands for mean square. Fs refers to the test value of F. R^2^ is the degree of interpretation of sample differences in different groups, that is, the ratio of group variance to total variance.

**Table 3:** Differences in microbiota community structures among three groups by AMOVA.

GroupVS	*SS*	*Df*	*MS*	*Fs*	*P*
N-O-F	0.605471	2	0.302736	1.28936	0.106
N-O	0.221809	1	0.221809	0.962758	0.474
N-F	0.406392	1	0.406392	1.71472	0.018*
O-F	0.281629	1	0.281629	1.18916	0.237

**Notes:**

* *P* < 0.05 asterisks indicate statistical significance.

SS is the total variance, also known as Sums of squared deviations. Df represents degree of freedom. MS stands for mean square. Fs refers to the test value of F.

### Taxonomic composition of fecal bacterial communities

The dominant six bacterial phyla of gut microbiota constituted the Bacteroidetes, Firmicutes, Proteobacteria, Verrucomicrobia, Fusobacteria and Actinobacteria (Fig. S1A). Bacteroidetes accounted for the highest proportion, contributing 56.02%, 57.04% and 51.21% of gut microbiota in the normal weight, overweight and obese groups, respectively. The Firmicutes were second contributing 37.25%, 35.02% and 36.79%, respectively. Proteobacteria, Verrucomicrobia, Fusobacteria, as well as Actinobacteria constituted the next most abundant phyla. Microbial compositions exhibited high interindividual variability, of which Bacteroidetes accounted for 21.23–75.21%, and Firmicutes accounted for 16.60–59.15% of all individuals.

According to the relative abundance analysis at the genus level, the fecal microbiome was predominated by sixteen genera: *Bacteroides*, *Faecalibacterium*, *Sutterella*, *Prevotella*, *Parabacteroides*, *Lachnospira*, *Phascolarctobacterium*, *Oscillospira*, *Dialister*, *Roseburia*, *Megamonas*, *Haemophilus*, *Fusobacterium*, *Escherichia*, *Ruminococcus*, and *Akkermansia*. The heatmap showed that, *Parabacteroides* (*F* = 1.032, *P* = 0.360), *Dialister* (*χ*^*2*^ = 2.469, *P* = 0.291) and *Ruminococcus* (*F* = 1.478, *P* = 0.233) exhibited a decreasing trend among the three groups, while *Prevotella* (*F* = 0.271, *P* = 0.763), *Lachnospira* (*F* = 1.691, *P* = 0.190) and *Megamonas* (*F* = 1.072, *P* = 0.369) displayed an increasing trend (Fig. S1B). Regrettably, these figures did not achieve statistical significance. However, *Oscillospira* (*χ*^*2*^ = 10.062, *P* = 0.001) decreased among the three groups, and *Sutterella* (*F* = 4.052, *P* = 0.020) increased.

### LEfSe analysis of phylogenetic and taxonomic profiles

**Figure 4:**
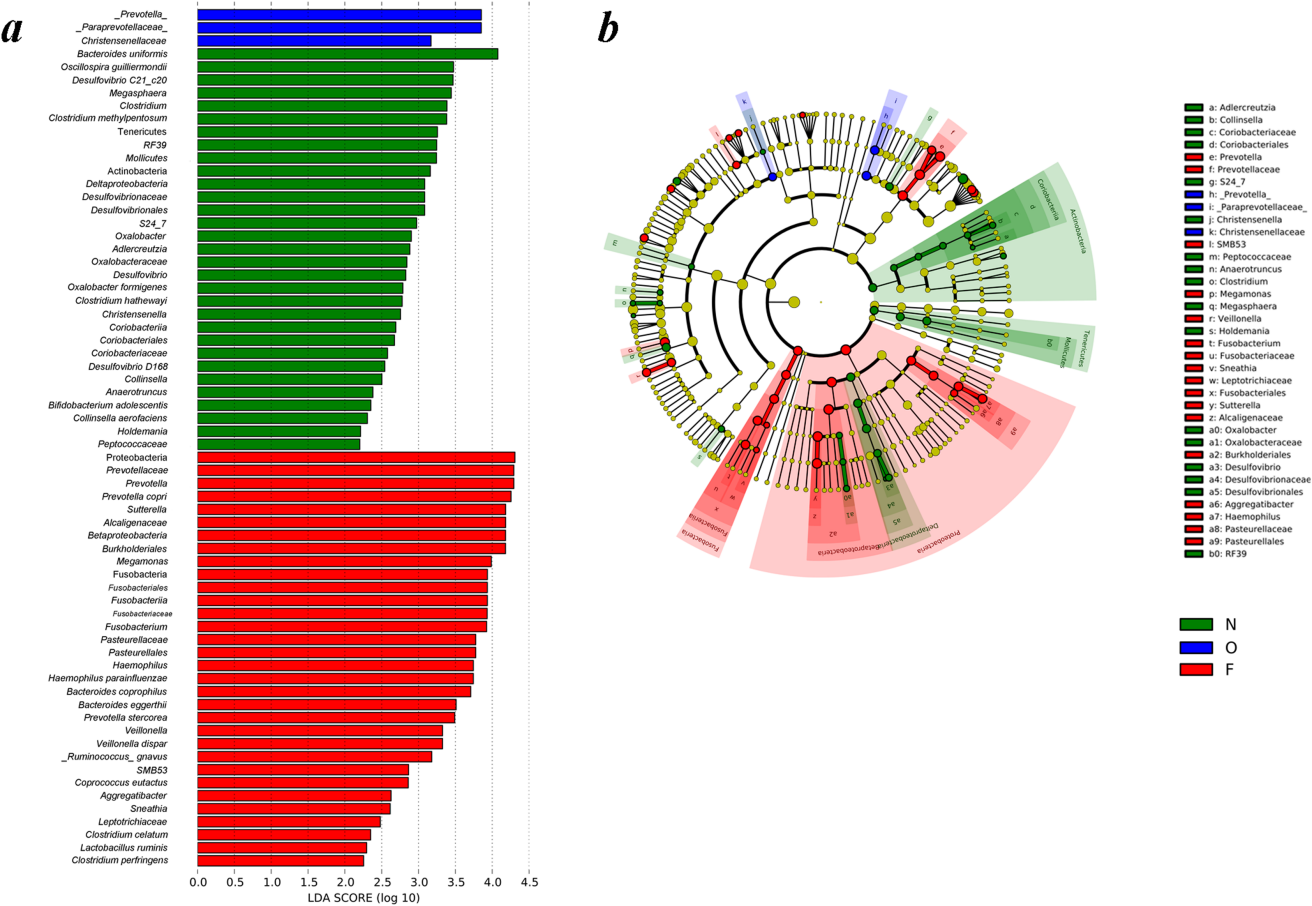
Different structures of gut microbiota in three groups. (A) Differences of the gut microbiota in the normal weight, overweight and obese groups were examined by LEfSe analysis. (B) Cladogram indicating the phylogenetic distribution of microbial lineages correlated with the three groups (the levels indicate, from the inner to outer rings, genus, family, order, class and phylum). (N, normal weight group; O, overweight group; F, obese group).

Differences of gut microbiota in the normal weight, overweight, as well as obese groups were examined by LEfSe analysis. The taxa, which had an alpha value of 0.05, as well as the absolute LDA (log10) scores > 2.0 were the only ones regarded significant. Thirty-one bacteria species were enriched in the fecal samples of the normal weight group, three in the overweight group, and 32 in the obese group. As shown in Fig. 4A, multiple genera were present in remarkably higher abundances in the gut microbiome of the normal weight group. These included *Megasphaera*, *Clostridium*, Oxalobacter, *Desulfovibrio*, *Christensenella*, *Collinsella*, *Anaerotruncus* and Holdemania. The genera *Prevotell*a, *Sutterella*, *Megamonas*, *Fusobacterium*, *Haemophilus*, *Veillonella*, *SMB53*, *Aggregatibacter* and *Sneathia* were abundant in the obese group. The LEfSe analysis generated cladogram exhibited the most differentially abundant taxa enriched in gut microbiota from three groups. The obese group exhibited a remarkable decrease in the Actinobacteria and Tenericutes phyla and a greater abundance of Proteobacteria and Fusobacteria relative to the normal weight group (Fig. 4B).

## Discussion

The importance of gut microbiota to obesity prevalence and development has been widely acknowledged. Early studies found that gut microbiota mainly consist of Firmicutes, Bacteroidetes, Proteobacteria, Actinobacteria, Verrucomicrobia and Fusobacteria, with Firmicutes and Bacteroidetes accounting for the majority (Eckburg et al., 2005). To be precise in this study, we ranked six phyla by proportion in gut microbiota of children and identified Bacteroidetes, Firmicutes, Proteobacteria, Verrucomicrobia, Fusobacteria, as well as Actinobacteria, with Firmicutes and Bacteroidetes accounting for 90%, which is consistent with our previous findings (Xiaowei et al., 2020). Some studies documented that the number of Bacteroidetes decreased while that of Firmicutes increased in people with obesity, and the ratio of Bacteroidetes to Firmicutes was significantly lesser in people with obesity than in people of normal weight (Ley et al., 2005; Patrone et al., 2016; Turnbaugh et al., 2008). Nevertheless, other studies concluded that there were no obvious differences in the ratio of Bacteroidetes to Firmicutes among individuals of different body weights (Karlsson et al., 2012; Schwiertz et al., 2010). Herein, we did not observe a remarkable difference in Firmicutes abundance, as well as the ratio of Firmicutes and Bacteroidetes among the three groups, while the percentage of Bacteroidetes in the obese group was less in contrast with that of the normal weight and overweight groups. Our previous study also found that the Firmicutes abundance and the ratio of Firmicutes and Bacteroidetes between the normal weight and obese groups had no statistical difference (Xiaowei et al., 2020). However, Ismail et al. (2011) found that distribution of Firmicutes and Bacteroidetes was remarkably increased in the children with obesity in contrast with the children of normal weight. Maya-lucas et al. found that some phyla Firmicutes and Bacteroidetes members were at least 2-fold more remarkably abundant in the children of normal weight relative to the children with obesity (Maya-Lucas et al., 2018). Hou et al. found that the relative richness ratio of Firmicutes and Bacteroidetes in the children with obesity was remarkably elevated in contrast with the children of normal weight (Hou et al., 2017). This difference might be explained by diet, region, nationality and age of the study subjects. Research conclusions on this field are not consistent in the world. Thus, more related research is required to explore the relationship of the ratio of Firmicutes and Bacteroidetes with obesity in children. Although differences can be found in studies on the gut microbiota diversity, findings agreed that the abundance of the gut microbiota in people with obesity is lower relative to that in people of normal weight (Cotillard et al., 2013; Le Chatelier et al., 2013). Kong et al. claimed that weight loss could increase the gut microbiota abundance, indicating that the abundance in obese individuals was lower in contrast with that in normal weight individuals, which might be simply reversed by reducing body weight (Kong et al., 2013). Our study also suggested that the abundance of gut microbiota decreased remarkably with increased body weight, and individuals with low microbial abundance had more marked overall adiposity. Similarly, the diversity of gut microbiota decreased significantly with the increased body weight, which was well consistent with related research results (Karlsson et al., 2012).

To further study the differences of gut microbiota in children, LEfSe analysis on the basis of the richness of OTUs was performed to screen key biomarkers (Wu et al., 2017). In the analysis of gut microbiota abundance with significant differences in the normal group, we observed a sharp increase in *Oscillospira guilliermondii*, *Clostridium*, *Bacteroides uniformis* and other 28 species of bacteria. A recent study in Canada documented that the *Oscillospira* richness, which was strongly linked with the reduction in childhood obesity, escalated in infant gut microbiota at three months old or when the expectant mother came into contact with pet animals (Tun et al., 2017). Wang et al. (Yan et al., 2012; Zhang et al., 2013) chronicled that relative to the control group, the number of *Lactobacillus* and *Bifidobacterium* decreased significantly, while Bacteroidetes and *Clostridium* exhibited an increasing pattern, with the latter increasing more significantly. *Clostridium* can increase the expression of GLUT2 in jejunum mucosa and the expression of fatty acid transferase in ileum mucosa, which can result in increased absorption of carbohydrates and fat (Anni et al., 2014). Cano et al. suggested that *Bacteroides uniformis* could reduce the weight of obese mice triggered by a high-fat diet (Gauffin Cano et al., 2012). The overall findings indicated that decreases in such gut microbiota would probably lead to pathogen increase, metabolic disorders, an imbalance of gut microecology, and in the end, obesity.

In the obese group, increases could be observed in *Prevotella*, *Bacteroides coprophilus*, *Bacteroides eggerthii*, *Ruminococcus gnavus*, *Lactobacillus ruminis, Perfringens* and other twenty-six species of bacteria. A comparative study in children from Europe, as well as rural Africa revealed that a large number of *Prevotella* existed in the gut microbiota of African children (plant-based diet). *Prevotella* can decompose dietary fiber and xylan to produce short chain fatty acids (De Filippo et al., 2010). In our study, a high proportion of *Prevotella* was detected in the gut microbiota in Chinese children owing to the similar diet structure with African children rather than European children. The results showed that diet was one of the important factors affecting gut microbial communities, and the diet resulted in great differences in gut microbial composition, structure and function among different populations. Gao et al. indicated that a significantly increased abundance of Fusobacterium was observed in people with obesity (Gao et al., 2018). Torres et al. reported that *Bacteroides coprophilus* increased in women with polycystic ovary syndrome, which was usually accompanied by obesity (Torres et al., 2018). Dziarski et al. summarized that *Bacteroides eggerthii* enhanced colitis (Dziarski et al., 2016). Previous studies revealed that *Ruminococcus gnavus* plays a vital role in inflammatory bowel disease and metabolic disorders (Le Chatelier et al., 2013; Peterson et al., 2015). Recent studies found that *Ruminococcus gnavus* in the obese group was more relative to that in the normal weight group (Liu et al., 2017). Additionally, Blanton et al. discovered that *Ruminococcus gnavus* in malnourished mice was less than that in nourished mice (Blanton et al., 2016). Thus, it was almost certain that *Ruminococcus gnavus* was positively related to BMI. O’Donnell et al. pointed out that *Lactobacillus ruminis* increased as the carbohydrate intake increased (O’Donnell et al., 2011). Hao et al. studied the bacteria in the feces of people with obesity in China. The findings demonstrated that relative to people having normal weight, the number of *Escherichia coli*, *Lactobacillus* and *Bifidobacterium* in people with obesity slightly decreased, while the numbers of *Perfringens* and *Bacteroides* were significantly decreased (Zuo et al., 2011). Overall, the significantly increased species of gut microbiota in the obese group was on the rise in various diseases, and it was believed that the pathogenic bacteria or opportunistic pathogens in the obese group increased.

In our previous study, UniFrac analysis was used to compare the similarity in the gut microbial communities and uncovered an evident clustering pattern in the normal weight group and the obese group. We found that the normal weight group and the obese group were distinct from each other in terms of gut microbiota composition (Xiaowei et al., 2020). Hou et al. found that the fecal microbiota from the group of children obesity could be separated from those of the control group via principal coordinate analysis (Hou et al., 2017). The same results were obtained in this study. Moreover, we found that the overweight group partially overlapped both the normal weight group and the obese group, from which it was assumed that the overweight individuals were likely to develop into either normal weight or obesity. On the other hand, only three bacteria species were identified in the overweight group, which may be explained by the overlaps with both the normal weight group and the obese group. We are planning to conduct an in-depth study to obtain a more comprehensive understanding of overweight children. Children who are overweight could either become obese or return to a normal weight. We hope that the in-depth study of this group can help more overweight children return to a normal weight and thus prevent the prevalence of obesity.

The current study has some limitations that should be considered. The present sample size may be insufficient, and further verification on a larger sample size is required. It was performed in cross-section and could not yield evidence of a causal effect between gut microbiota and obesity. Although the inclusion, as well as exclusion criteria, was set, the findings were affected by the factors of the participants themselves, like their diets and physical activities, which might have an impact on gut microbiota. We did not measure data of metabolic deregulation related with obesity, such as, serum levels of glucose, high-density lipoprotein cholesterol, insulin, triglycerides, low-density lipoprotein cholesterol, as well as total cholesterol. The participants recruited in this study only represented the composition of gut microbial of children in one distinct region. Gut microbial samples in this study were all collected at one certain point-in-time.

## Conclusions

Herein, we found that in a group of Chinese children, the abundance of species decreased significantly as the body weight increased. The gut microbial composition between children of normal weight and obesity was notably different, but gut microbiota in overweight children showed similarities to that of children of normal weight and obesity. Based on this, an in-depth study is needed to obtain a more comprehensive understanding of children who are overweight, a special group between normal weight and obesity. Exploring the role of specific gut microbial species could improve the individualized targeted gut microbial therapy for overweight children and obesity. We hope that this study could serve as a basis for future in-depth analyses on the overweight group, with an objective of helping more overweight children return to normal weight and thus preventing the prevalence of obesity.

## Supplemental Information

10.7717/peerj.11439/supp-1 Supplemental Information 1 Taxonomic composition of fecal bacterial communities. (a) Histogram of the community composition of gut microbiota at the phylum level. The abscissa represents the group, and the ordinate represents the relative abundance. (b) Heatmap diagram of the gut microbiota composition for the 30 most abundant OTUs classified by genus. Heatmap depicting the distribution and relative abundance. (N, normal weight group; O, overweight group; F, obese group).

10.7717/peerj.11439/supp-2 Supplemental Information 2 Body mass index cut-offs for overweight and obesity in Chinese children and adolescents aged 2–18 years.

10.7717/peerj.11439/supp-3 Supplemental Information 3 The raw sequence data for all samples used in this study.

10.7717/peerj.11439/supp-4 Supplemental Information 4 Raw demographic data.

## Additional Information and Declarations

### Data Availability

The following information was supplied regarding data availability: The raw sequence data are available at the Genome Sequence Archive at the BIG Data Center, Beijing Institute of Genomics (BIG), Chinese Academy of Sciences: CRA002887: https://bigd.big.ac.cn/bioproject/browse/PRJCA002952
