# Supplementary figures and images for: Characterization of the gut microbiota in Chinese children with overweight and obesity using 16S rRNA gene sequencing
Source: PeerJ. 2021 Jun 8;9:e11439. doi: 10.7717/peerj.11439 (PMC8194416; doi:10.7717/peerj.11439)

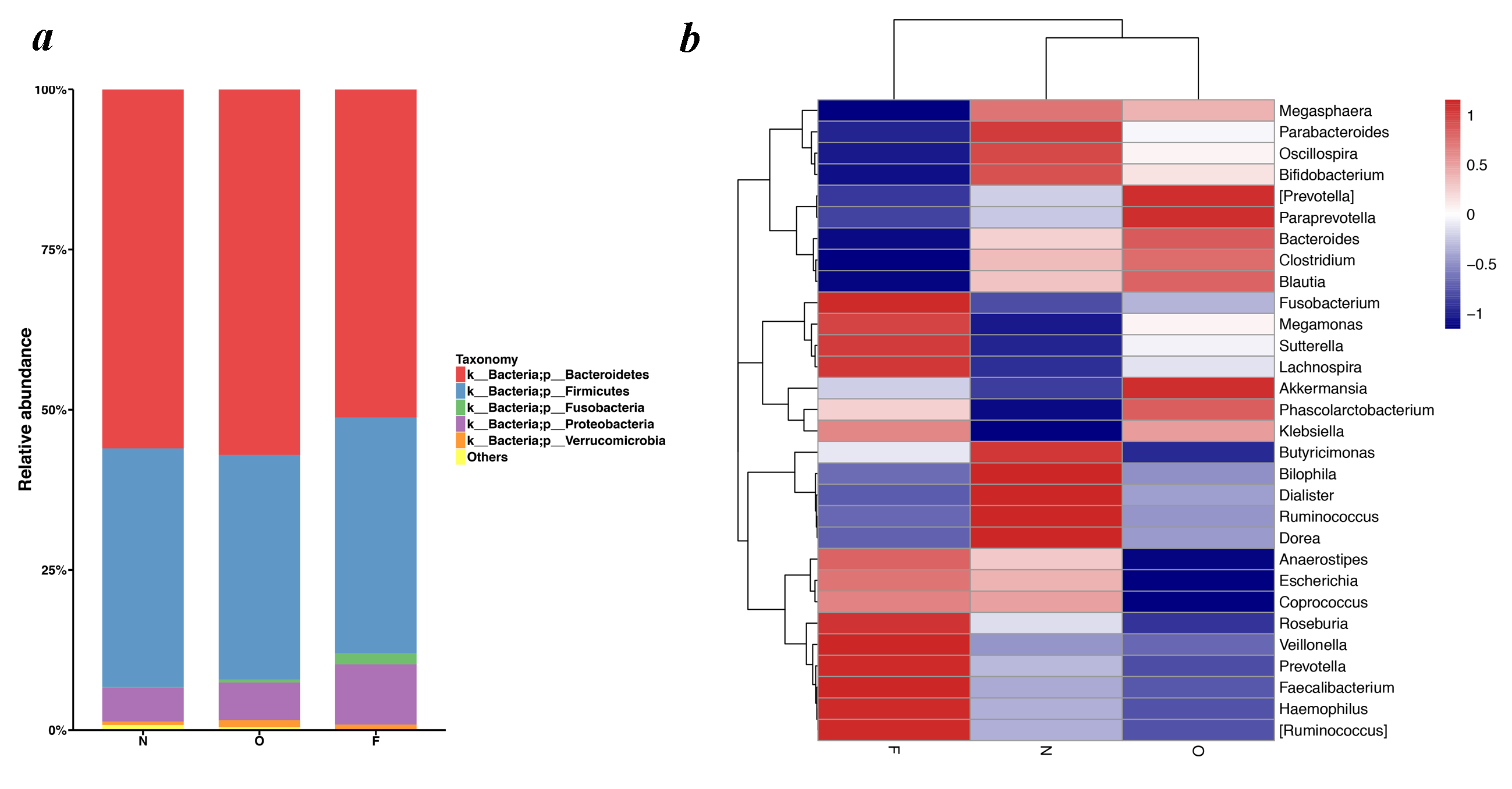

Supplement: Supplemental Information 1 — (a) Histogram of the community composition of gut microbiota at the phylum level. The abscissa represents the group, and the ordinate represents the relative abundance. (b) Heatmap diagram of the gut microbiota composition for the 30 most abundant OTUs classified by genus. Heatmap depicting the distribution and relative abundance. (N, normal weight group; O, overweight group; F, obese group). [file peerj-09-11439-s001.png]
