# Supplementary material for: Characterization of the gut microbiota in Chinese children with overweight and obesity using 16S rRNA gene sequencing
Source: PeerJ. 2021 Jun 8;9:e11439. doi: 10.7717/peerj.11439 (PMC8194416; doi:10.7717/peerj.11439)
Supplement: Supplemental Information 2 [file peerj-09-11439-s002.docx]

Supplementary Table S1 Body mass index cut-offs for overweight and obesity in Chinese children and adolescents aged 2-18 years

| Age (Years) | Boys | | Girls | |
| --- | --- | --- | --- | --- |
|  | Overweight | Obesity | Overweight | Obesity |
| 2 | 17.5 | 18.9 | 17.5 | 18.9 |
| 3 | 16.8 | 18.1 | 16.9 | 18.3 |
| 4 | 16.5 | 17.8 | 16.7 | 18.1 |
| 5 | 16.5 | 17.9 | 16.6 | 18.2 |
| 6 | 16.8 | 18.4 | 16.7 | 18.4 |
| 7 | 17.2 | 19.2 | 16.9 | 18.8 |
| 8 | 17.8 | 20.1 | 17.3 | 19.5 |
| 9 | 18.5 | 21.1 | 17.9 | 20.4 |
| 10 | 19.3 | 22.2 | 18.7 | 21.5 |
| 11 | 20.1 | 23.2 | 19.6 | 22.7 |
| 12 | 20.8 | 24.2 | 20.5 | 23.9 |
| 13 | 21.5 | 25.1 | 21.4 | 25.0 |
| 14 | 22.1 | 25.8 | 22.2 | 25.9 |
| 15 | 22.7 | 26.5 | 22.8 | 26.7 |
| 16 | 23.2 | 27.0 | 23.3 | 27.2 |
| 17 | 23.6 | 27.5 | 23.7 | 27.6 |
| 18 | 24.0 | 28.0 | 24.0 | 28.0 |
