# Supplementary material for: Characterization of the gut microbiota in Chinese children with overweight and obesity using 16S rRNA gene sequencing
Source: PeerJ. 2021 Jun 8;9:e11439. doi: 10.7717/peerj.11439 (PMC8194416; doi:10.7717/peerj.11439)
Supplement: Supplemental Information 3 [file peerj-09-11439-s003.docx]

The raw sequence data reported in this paper have been deposited in the Genome Sequence Archive at the BIG Data Center, Beijing Institute of Genomics (BIG), Chinese Academy of Sciences, under accession number CRA002887, which is publicly accessible at <https://bigd.big.ac.cn/gsa>.
